# Unraveling Circular and Messenger RNA Dynamics in Colorectal Tumorigenesis: Insights into Tissue Heterogeneity and MSI-MSS Tumor Distinction

**DOI:** 10.3390/genes17091091

**Published:** 2026-09-10

**Authors:** Sabine Vautier, Corentin Levacher, Florent Marguet, Edwige Kasper, Jean-Christophe Sabourin, Stéphanie Baert-Desurmont, Philippe Ruminy, Claude Houdayer

**Affiliations:** 1 Department of Genetics, CHU Rouen, Inserm U1245, Univ Rouen Normandie, Normandie Univ, F-76000 Rouen, France; sabine.vautier@chu-rouen.fr (S.V.); edwige.kasper@chu-rouen.fr (E.K.); s.baert-desurmont@chu-rouen.fr (S.B.-D.); 2 Inserm U1245, Univ Rouen Normandie, Normandie Univ, F-76000 Rouen, France; corentin.levacher2@univ-rouen.fr; 3 Department of Pathology, CHU Rouen, Inserm U1245, Univ Rouen Normandie, Normandie Univ, F-76000 Rouen, France; florent.marguet@chu-rouen.fr (F.M.); jean-christophe.sabourin@chu-rouen.fr (J.-C.S.); 4 Centre Henri Becquerel, Inserm U1245, Univ Rouen Normandie, Normandie Univ, F-76000 Rouen, France; philippe.ruminy@chb.unicancer.fr

**Keywords:** circular RNA, colorectal cancer, MSI, MSS

## Abstract

Background/Objectives: Circular RNAs (circRNAs) are emerging regulators of genetic information that share the spliceosome biogenesis pathway with messenger RNAs (mRNAs), influencing their expression. There is an increasing body of evidence supporting their role in colorectal cancer (CRC) tumorigenesis. This study explored circRNAs in two distinct CRC tumorigenesis pathways: microsatellite instability (MSI) and microsatellite stability (MSS). We investigated competition between mRNA and circRNAs from their host genes, which could potentially disrupt normal gene regulation, and examined specific patterns of alterations in MSS and MSI tumors. Methods: Circular (circ) and linear (lin) exon–exon junctions were quantified using exon-specific probes targeting 48 genes involved in CRC predisposition and tumorigenesis processes. RNA was extracted from colorectal FFPE samples (stage 1 to 4 adenocarcinomas and adenomas). MSS tumors (MSS-TTs) and adjacent normal tissue (MSS-NT) were selected from 21 patients with a severe personal or family history of cancer. MSI tumors (MSI-TTs) and adjacent normal tissue (MSI-NT) were selected from 16 patients. Muscle content was also investigated as a potential confounding factor. Results: Principal component analysis distinguished NT from TT samples based on the sums of circular and linear junctions. CircRNA abundance was higher in samples with an elevated muscle content (Kruskal–Wallis test *p*-value = 0.034). Linear regression, adjusted for muscle content, showed significantly reduced global circRNA levels in tumors compared to in healthy tissues (MSI and MSS combined, *p*-value = 0.00268). In the MSS group, significant differences were observed between MSS-NT and MSS-TT in terms of circ/lin ratios and linear and circular junction counts for specific genes. MSI analysis revealed distinct gene profiles, with significant differences only in linear junction counts. Conclusions: Our results do not suggest competition between the circRNAs and mRNAs of the key oncogenic genes that we investigated, but they do reveal differences in circRNA/mRNA expression patterns within normal and tumor tissues.

## 1. Introduction

Colorectal cancer (CRC) is one of the leading causes of cancer worldwide, ranking third in terms of incidence among both men and women in 2022 [1]. Advances in high-throughput RNA sequencing have highlighted the importance of the transcriptome in revealing the molecular heterogeneity of CRC and improving our understanding of the disease. Recently, circular RNAs (circRNAs) have gained attention due to their unique and intriguing features. These covalently closed single-stranded RNA sequences, which are generated by backsplicing, have the same pre-messenger RNA (pre-mRNA) precursors as messenger RNA (mRNA) [2]. These molecules are particularly stable and exhibit tissue-specific and cell-specific expression patterns [3]. Recent studies have emphasized their significance as regulatory factors in tumor gene expression and pathological networks, contributing to tumor growth, metastasis, and drug resistance [4]. In CRC, a reduction in circRNA levels has recently been proposed as a key feature in the early stages of colorectal carcinogenesis [5]. Several functions of circRNAs have been identified, including their roles as microRNA (miRNA) sponges and their interactions with RNA-binding proteins and transcriptional regulators [6]. Importantly, this transcriptional regulation may influence the expression of parental genes [7], and previous studies have shown disruption to the circRNA/mRNA ratio in tumor tissues [8], as well as the existence of a physiological balance between circRNA and mRNA that can be disrupted under pathological conditions [9].

In light of these considerations, we hypothesize that competition between mRNA and circRNAs from their host genes could disrupt the physiological balance and the proper regulation of genes, which could be a feature of colorectal tumorigenesis. Therefore, different alteration patterns should be observed depending on the colorectal carcinogenesis pathway.

To this end, we studied microsatellite instability (MSI) and microsatellite stability (MSS) in colorectal tissues, representing two distinct tumorigenesis pathways. Tumor tissues (TTs) were selected along with the adjacent normal tissues (NTs). To maximize the potential for discovery, the MSS tissues were not chosen at random, but were obtained from patients with a severe personal and/or family history of CRC, i.e., those suspected of having a predisposition to CRC. We also selected 48 genes involved in CRC predisposition and tumorigenesis processes. We determined their circRNA/mRNA levels using SEALigHTS (Splice and Expression Analyses by Exon Ligation and High-Throughput Sequencing), a method that allows the simultaneous exploration of splicing and backsplicing by studying all exon–exon junctions within a panel of genes using probes designed at exon boundaries [10]. Our results do not support the existence of competitive mechanisms between mRNA and circRNA, but they show distinct mRNA/circRNA profiles between tumor and normal tissues with different patterns according to microsatellite status.

## 2. Materials and Methods

### 2.1. Patient and Sample Selection

We carried out a retrospective search for patients suspected of being predisposed to CRC, who did not have any actual genetic diagnosis and met previously described criteria (Supplemental Table S1) [11]. We selected 21 patients with available FFPE (Formalin-Fixed Paraffin-Embedded) samples, including biopsies and surgical specimens. All samples had MSS (microsatellite-stable) tumor status, determined based on immunohistochemistry of the four MMR proteins and microsatellite analyses (Promega MSI analysis system, Promega, Madison, WI, USA), and we will refer to this first group as MSS hereafter. The second group of 16 patients was selected based on the MSI phenotype of their tumors. Among these patients, seven were diagnosed with Lynch syndrome, characterized by constitutional variations in the following genes: *MLH1* (one patient), *MSH2* (two patients), *MSH6* (two patients), and *PMS2* (two patients). Additionally, two patients exhibited somatic hypermethylation of the *MLH1* promoter, and two others presented with the p.V600E mutation in *BRAF* exon 15. In the remaining five patients, the MSI tumor phenotype could not be explained. Our collected FFPE samples included normal tissues, adenomas, and stage 1 to 4 adenocarcinomas from both patient groups and were obtained from the “*Centre de Ressources Biologiques institutionnel du CHU de Rouen*” biobank (application number MRCBi/2024/10). All patients signed informed consent for genetic analysis in diagnosis and research.

### 2.2. Muscle Content

Previous studies have highlighted that muscle tissue contains elevated levels of circular RNA [12]. In order to assess the influence of muscle content on our data, we estimated the proportion of muscle tissue in each sample and classified them into the following categories: <5%, 5–15%, 15–25%, 25–50%, and >50%. We assessed the circular RNA levels as a function of muscle content, independent of whether the samples were NT or TT. We also investigated the influence of muscle content using an additional approach. We selected 10 samples with a high proportion of muscle tissue. Double RNA extractions were performed for each sample: the first using the entire paraffin-embedded tissue and the second adjusting the extraction area to minimize the amount of muscle tissue included. Our goal was to compare overall circRNA levels between these two RNA extracts for each sample (Supplemental Table S2). In addition to the 48 cancer genes selected for the study (Table 3, Section 3), and to support our analysis of muscle content, we also targeted 14 circRNAs that are known to be highly expressed in colorectal tissue [13] (Table 1).

### 2.3. Sample Processing and RNA Extraction

An expert pathologist reviewed Hematoxylin–Eosin–Safran (HES)-stained slides, confirming the histopathological characteristics and selecting tumoral tissue (TT) and, where possible, normal tissue (NT) distant from the tumor. Tumor cellularity for TT was estimated using the following thresholds: <5%, 5–15%, 15–25%, 25–50%, and >50%, and samples with less than 15% tumor content were excluded. RNA extraction was performed for 81 samples using the Maxwell^®^ 16 AS3000 instrument and the Maxwell^®^ 16 LEV RNA FFPE Kit (Promega Corporation, Madison, WI, USA), following the manufacturer’s recommendations and including the optional DNAse digestion step.

### 2.4. SEALigHTS Assay

The SEALigHTS method has been described in detail elsewhere [10] and allows the simultaneous analysis of all exon–exon junctions in a panel of genes of interest thanks to probes designed at exon extremities. Following reverse transcription and probe hybridization on cDNA, nearby probes are ligated if splicing and/or backsplicing occurs, and the number of ligations is quantified using unique molecular identifiers (UMIs) and high-throughput sequencing. All possible combinations of exons, i.e., splicing and backsplicing, are detectable. We used a panel comprising 2160 probes, specific to the exon extremities of 48 genes involved in cancer predisposition and tumorigenesis. These genes were defined by expert groups from the French scientific society “*Groupe Génétique et Cancer*”, which selected a number of key genes for use in diagnosis and translational research. A complete list of the targeted genes is available in the Section 3 (Table 3). Each junction is quantified using a custom Python 3.13.3 script, which processes FASTQ files to generate UMI count matrices that combine all patients and detected junctions (the script is available at https://github.com/U1245/CircRNA_CRC_SEALigHTS, accessed on 26 August 2026). To compare the abundance of each junction across samples, we normalized the UMI counts for each junction relative to the total UMIs in the corresponding sample.

$$Panel\;junction\;UMI\;normalized\;junction=\frac{UMI\;junction}{\sum UMI\;of\;all\;junctions\;from\;the\;panel\;for\;the\;sample}$$

From this point forward, UMI counts will always refer to the normalized counts.

For each gene and sample, we calculated the total number of UMIs for both circular and linear junctions and then determined the ratio between these types of junctions. As circular junctions represent specific individual circRNAs, their global abundance per gene was estimated using the sum of the UMI count of circular junctions. For mRNAs, we estimated abundance by dividing the sum of the UMI count of a gene’s linear junctions by the number of exons minus one.

$$mRNA\;abundance\;for\;one\;gene=\frac{\sum UMI\;linear\;junction\;for\;the\;gene}{Number\;of\;exons-1}$$

### 2.5. Statistical Analysis

All statistical analyses were conducted using R, version 4.4.0.

### 2.6. Muscle Content Analysis

A Kruskal–Wallis test was applied to the sums of circular junctions to evaluate potential imbalances across the five muscle content categories. In addition, a two-sided Wilcoxon rank-sum test with Benjamini–Hochberg False Discovery Rate (FDR) correction for multiple testing was performed to identify specific differences between the muscle content categories. A two-sided Wilcoxon signed-rank test was also applied to 10 pairs of extraction duplicates, focusing on circular junctions from the gene panel.

### 2.7. CircRNA Analysis

We compared the NT and TT groups based on the sums of all circRNAs from the gene panel using a two-sided Wilcoxon rank-sum test, followed by linear regression adjusted for muscle content. We compared the TT and NT groups, as well as the MSI and MSS groups, across various parameters, including the UMI counts of specific circRNAs, the sums of circular and linear mRNA junction UMI counts, and the circular junction/linear mRNA junction ratio (circ/lin ratio) per gene. We performed linear regression adjusted for muscle content with FDR correction between groups.

An alpha threshold of 0.05 was applied in all statistical analyses performed.

Throughout the manuscript, linear junctions (lin) correspond to mRNA.

## 3. Results

### 3.1. Muscle Content Is a Confounding Factor in circRNA Analyses

**Figure 1:**
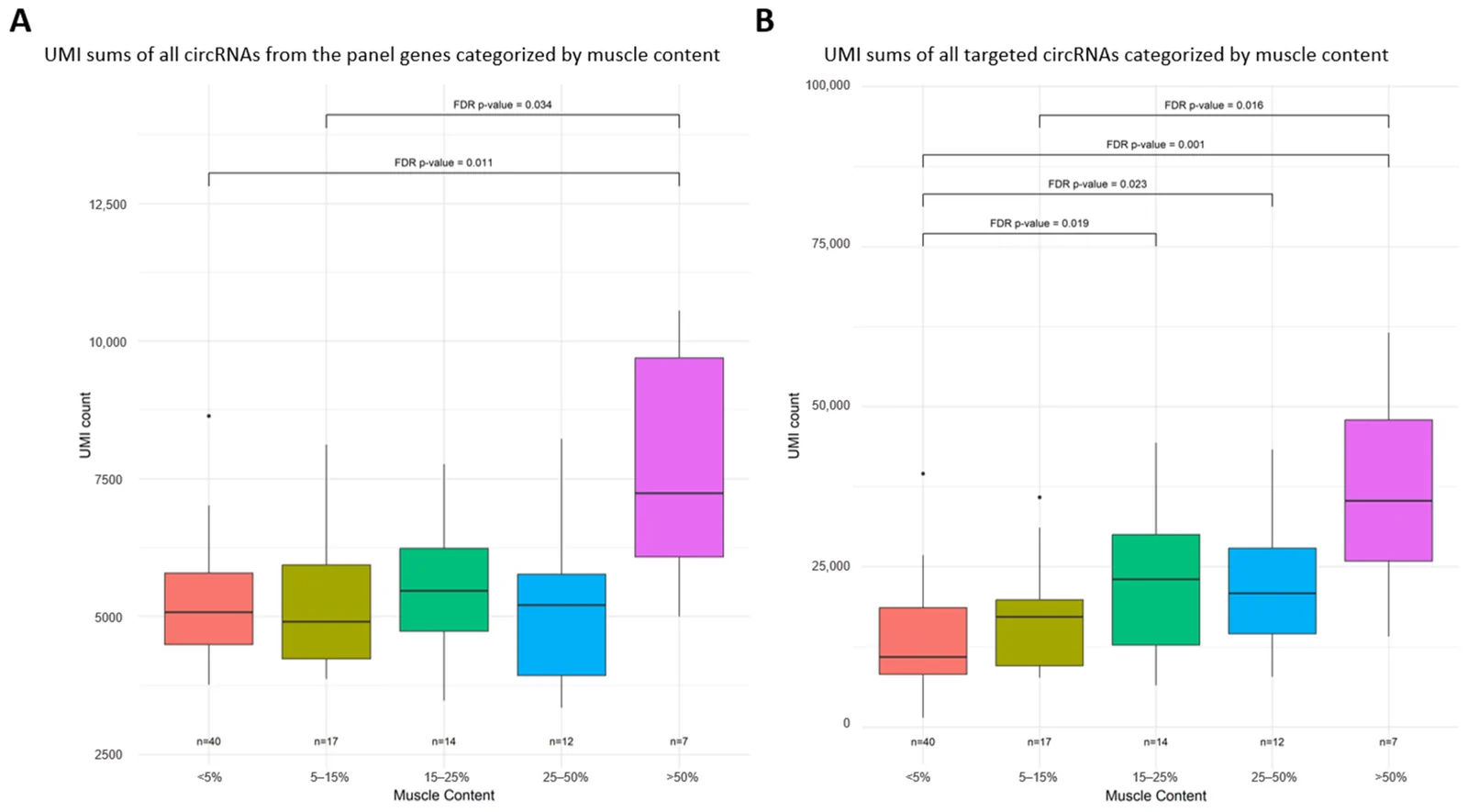
Analyses of circRNA levels across five categories of muscle content. *X*-axis: muscle content, divided into 5 categories. *Y*-axis: UMI counts. (**A**) Results for the 48 genes selected for the study. (**B**) Results for the 14 targeted and highly expressed circRNAs.

**Figure 2:**
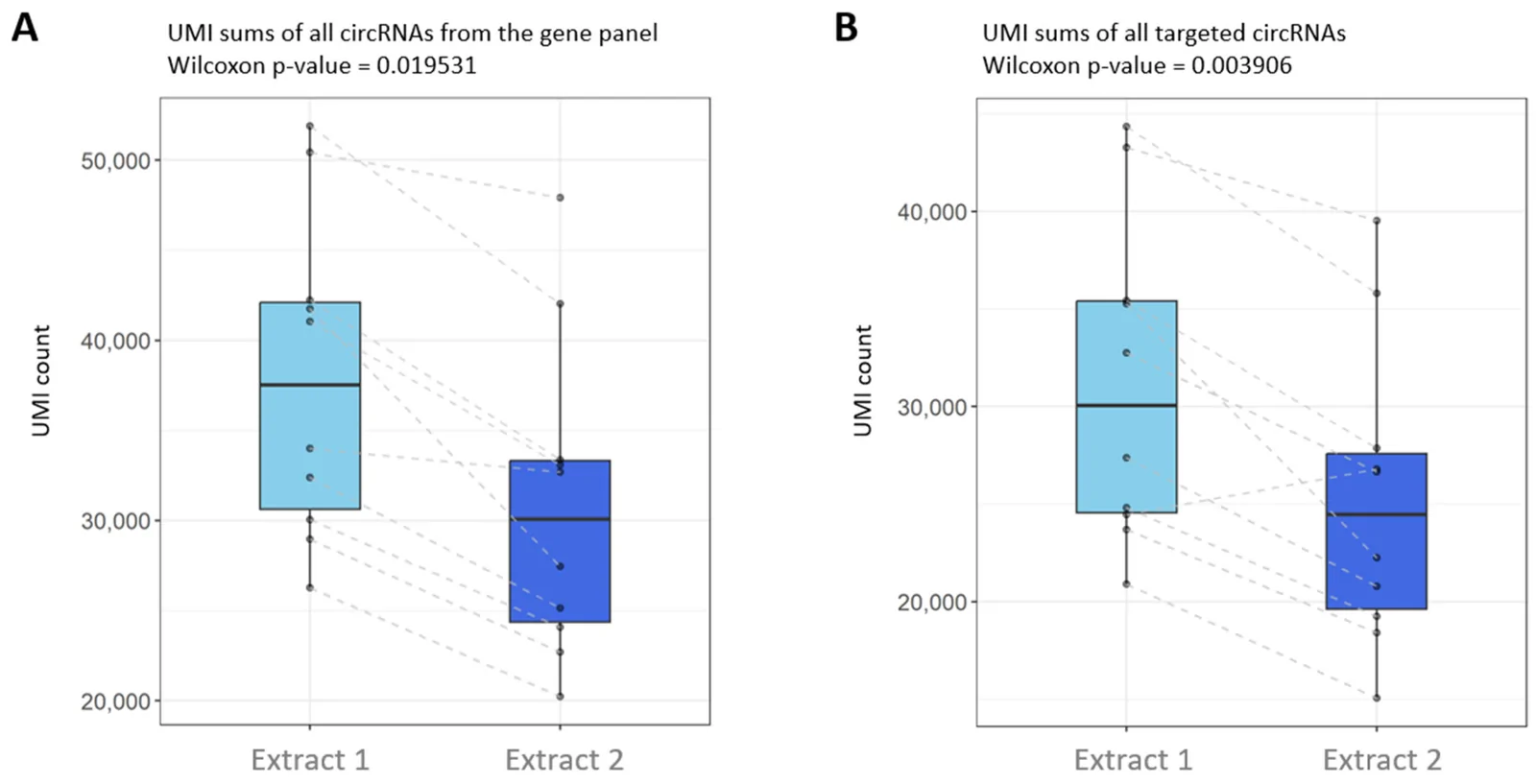
Analysis of circRNA levels in ten samples that underwent two different extractions. (**A**) Results for the 48 genes selected for the study. (**B**) Results for the 14 targeted and highly expressed circRNAs.

Sample analysis according to the five muscle content categories revealed a significant difference in the total sum of UMIs for the circular junctions of all genes between categories, as identified by the Kruskal–Wallis test (*p*-value = 0.034). The Wilcoxon test further highlighted significant differences between groups <5% and >50%, as well as between groups 5–15% and >50% (FDR-adjusted *p*-values of 0.011 and 0.034, respectively) (Figure 1A). The Kruskal–Wallis test also revealed a significant difference between groups for the sum of the UMIs for the targeted circRNAs highly expressed in colorectal tissue (*p*-value = 0.0003) (Figure 1B). The Wilcoxon test further specified that these differences were significant between the following groups: <5% vs. 15–25%, 25–50%, and >50%, and 5–15% vs. >50% (FDR-adjusted *p*-values of 0.0189, 0.0235, 0.0011, and 0.0163, respectively). These data show that muscle content significantly increases overall circRNA levels. We then analyzed the samples that underwent double extraction. Samples from the second extraction, which used less muscle tissue, showed a significant reduction in the sum of all UMIs for circular junctions compared to those from the first extraction, i.e., the extraction using all of the tissue (*p* = 0.0195) (Figure 2). Consequently, to remove bias linked to high muscle content, we excluded seven RNA extracts from samples containing over 50% muscle tissue, as this category exhibited the most pronounced differences. We then applied a linear regression model to compare the different groups while accounting for the varying proportions of muscle tissue in each sample. We also removed the first extract of each double extraction, leaving 64 RNA samples for the rest of the study, i.e., 16 normal tissue samples and 13 tumor tissue samples for MSI patients, and 14 normal tissue samples and 21 tumor tissue samples for MSS patients (Table 2).

### 3.2. Splicing and Backsplicing Profiles Differ Between Normal and Tumor Tissues

**Figure 3:**
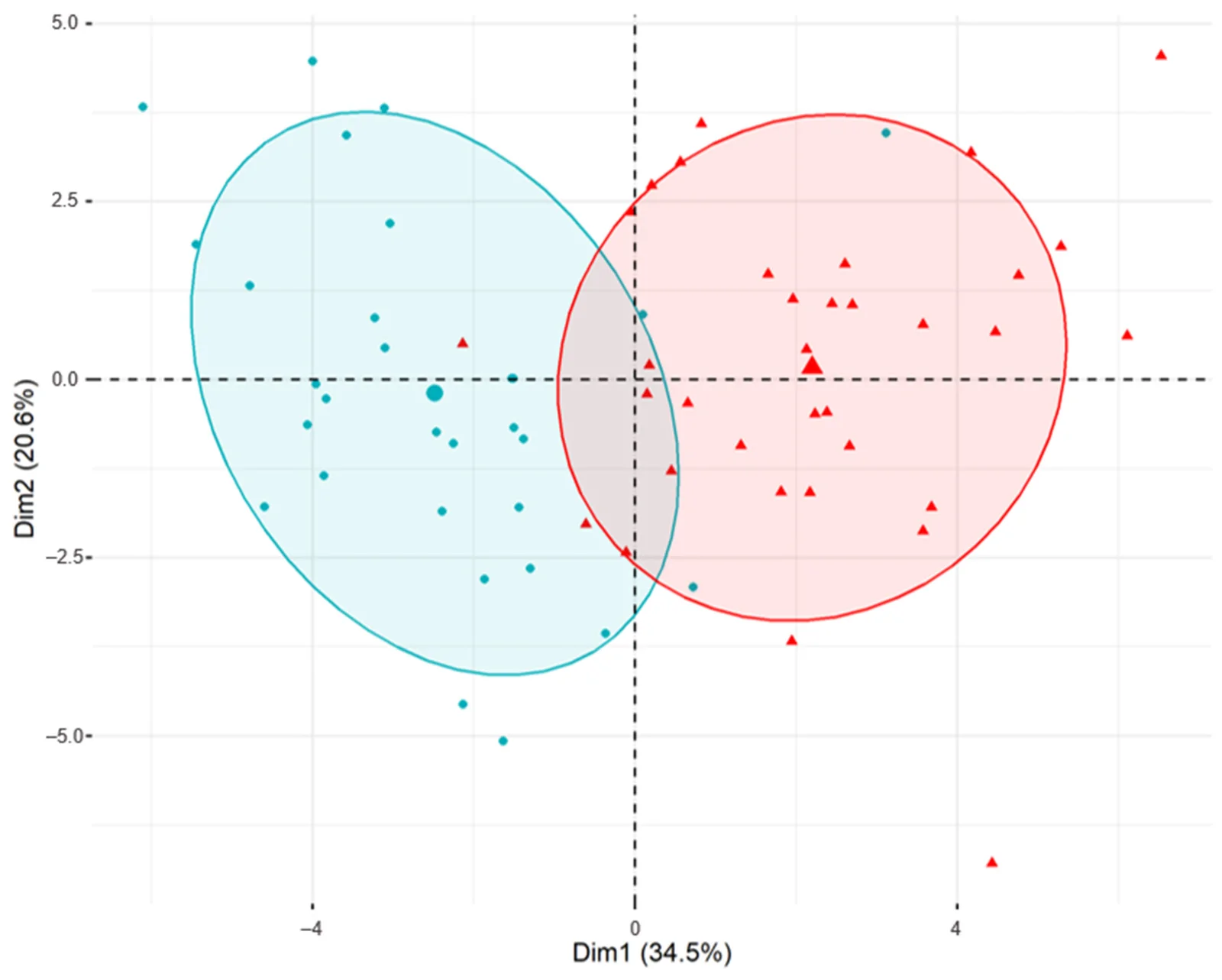
Principal component analysis based on the sum of circular and linear junction counts for each sample, using genes that showed differences in circRNA and mRNA expression between normal and tumor tissues. Blue circles: normal tissue samples. Red triangles: tumor tissue samples. Blue and red ellipses represent the 70% confidence interval for the normal and tumor groups, respectively.

SEALigHTS detected 359 circular and 1348 linear junctions (Table 3). A minimum of 20% mapping of left and right probes was deemed necessary to avoid primer dimers and ensure sufficient ligation and reliable results (Supplemental Table S3). All canonical junctions were correctly identified. A positive correlation was observed between exon number and the number of linear junctions (ρ = 0.767, *p* < 0.0001, Spearman’s rank correlation coefficient), as well as between the number of linear junctions and the number of circular junctions (ρ = 0.766, *p* < 0.0001). Comparing the total sums of linear junctions between the TT and NT groups provides an approximation of the differential expression of the corresponding mRNA (Supplemental Table S4). We then searched for differences in circRNA and mRNA expression between normal and tumor tissues. We performed principal component analysis (PCA) based on the sums of the circular and linear junction UMIs per sample, restricting it to genes with significant differential expression between normal and tumor tissues (Figure 3). The first component (PC1), which accounted for 34.5% of the total variance, enabled separation between the NT and TT samples, indicating distinct transcriptomic profiles. Together, PC1 and PC2 accounted for 55.1% of the overall variability.

### 3.3. Normal and Tumor Tissues Show Disequilibrium in circRNA Expression

**Figure 4:**
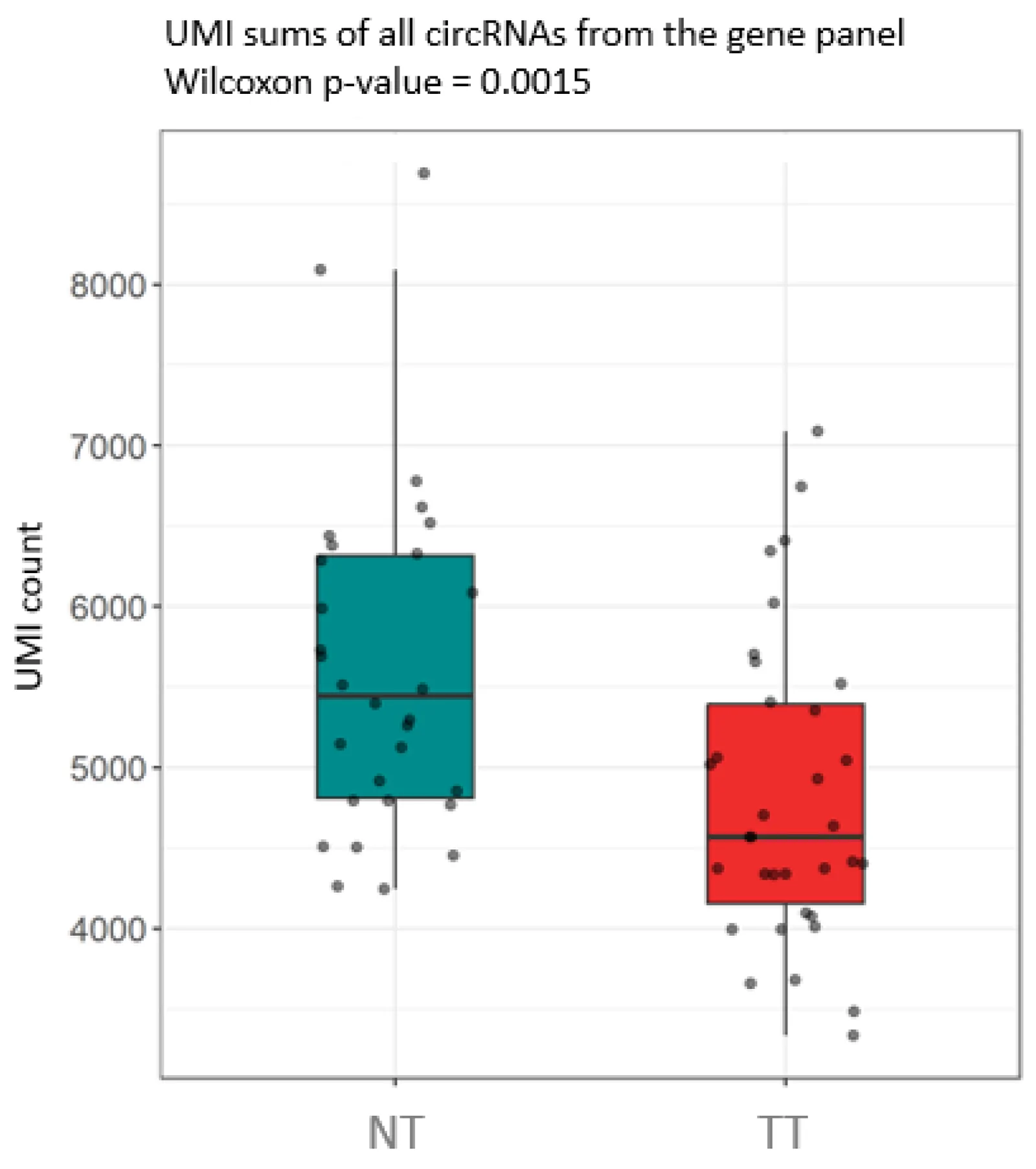
Reduction in the sum of total circRNA junctions between normal and tumor samples (NT and TT, respectively).

A two-sided Wilcoxon rank-sum test considering all circular RNAs revealed a significant reduction in circular junction content in TT compared to in NT (*p*-value = 0.0015), when the groups of 30 NT and 34 TT samples (including both MSI and MSS tumors) were compared (Figure 4). Linear regression analysis, adjusted for muscle content, confirmed this decrease in circular junction content (*p* = 0.00268).

### 3.4. Comparative Analysis of MSI and MSS Samples

A comparison of the 13 MSI tumor samples with the 21 MSS tumor samples showed no significant differences in terms of counts of linear and circular junctions or the circ/lin ratio at the gene level, based on linear regression adjusted for muscle content with FDR correction. Similarly, comparing these parameters between 16 MSI and 14 MSS normal tissue samples using the same approach showed no significant differences.

### 3.5. CircRNA/mRNA Expression Is Stable in MSI Samples

Focusing on the MSI group with 16 NT samples and 13 TT samples, differences were only observed in the normalized UMI counts of linear junctions, based on FDR-adjusted *p*-values from linear regression accounting for muscle content (Table 4). A significant increase in linear UMI counts was found for *AXIN2*, *RNF43*, *BRCA1*, *BUB1*, *CHEK2*, *BRCA2*, and *TP53*, while a significant decrease was observed for *GALNT12*, *CDH1*, and *APC*, reflecting differences in mRNA expression. No significant differences were identified in the circular junction sums or circ/lin ratios. This suggests that the variation in mRNA is offset by a proportional variation in circRNA, even if the latter is not statistically significant due to its low expression and high variability (Figure 4).

### 3.6. CircRNA/mRNA Expression Is Variable in MSS Samples

By contrast, a specific analysis of the MSS samples (14 NT and 21 TT samples) revealed significant differences in all three parameters studied between NT and TT, and five groups could be distinguished (Table 5). The first group of genes showed a decrease in the circ/lin UMI ratio explained by a significant decrease in the circular junction UMI counts (*FAM175A*, *POLD1*, *FAN1*, *MSH2*, and *MSH3*) or a significant increase in linear junction UMI counts (*AXIN2* and *RNF43*). The second group showed a significant decrease in all three parameters (*BMPR1A* and *ATM*) that could be explained by a greater proportional decline in circRNAs compared to mRNAs. The third group exhibited only a decrease in the circ/lin ratio (*MRE11*, *BRIP1*, *BRCA1*, *BARD1*, and *CHEK2*). This suggests that mRNA and circRNA levels vary slightly but consistently and not significantly. This may also be due to the high variability observed in the number of circular and linear junctions (see Figure 3 and Figure 4). The fourth group showed no significant variation in the circ/lin ratio, which could be explained by a simultaneous significant decrease in both circular and linear UMI counts (*APC* and *SMAD4)*, suggesting that the circRNAs and mRNAs of these genes are linked by a common mechanism. Lastly, the fifth group showed no significant variation in the circ/lin ratio despite a significant decrease in circRNA counts (*MBD4*, *RIC8B*, *KRAS*, *MLH1*, and *CCSER2*) or a significant increase in linear junctions (*XRCC2* and *NTHL1*).

## 4. Discussion

Although circRNAs are increasingly recognized as potentially important factors in prevention, diagnosis, and therapy, the complexity of their detection means that original data are limited in the field of oncology and are even extremely scarce regarding their role in MSS and MSI colorectal tumors. One previous study addressed the association between circRNAs and distinct colorectal subtypes, including MSI status, but the small sample size increased the risk of type II errors [15]. Consequently, to the best of our knowledge, this is the first original study to analyze the levels of circRNA and messenger RNA in MSI and MSS colorectal tumors using a sensitive and robust detection method. This was made possible thanks to SEALigHTS, which simplifies the identification of circular and linear junctions [10,16]. Some of the circular RNAs under study had previously been validated by RT-PCR [16], and, in this study, SEALigHTS successfully detected known circular RNAs (Table 1). Its high sequencing depth and use of normalized UMI counts, followed by statistical correction for muscle content (see below), ensure the accurate characterization and quantification of mRNA and circRNA isoforms. By relying on probe hybridization at exon boundaries, SEALigHTS circumvents the major limitations associated with degraded RNA from FFPE samples, as paraffin embedding degrades RNA, reducing its fragment size and causing sequence modifications [17]. The analysis of linear junction counts, used as a proxy for gene expression, is consistent with larger RNA-seq studies (Supplemental Table S4). Indeed, we compared our data with RNA-seq data from the NCBI Gene Expression Omnibus (GEO; GSE156451 [18]), which includes 47 colorectal tumors and 48 normal tissues, and with RNA-seq data from 1063 tumors and 120 normal colorectal tissues [19]. We found that the significant differences were consistent in terms of sensitivity and direction of variation for 24 of the 26 genes for which a comparison was possible. Moreover, PCA (Figure 3) recapitulated the transcriptomic differences between normal and tumor tissues using genes that exhibit differential circRNA and mRNA expression, illustrating the effectiveness of our approach.

Both messenger and circular RNAs exhibit tissue-specific expression [20,21], as well as specific alternative splicing [22]. Garcia-Rodriguez et al. showed that muscle tissue can interfere with bulk circRNA analysis, since the *muscularis propria* contains higher levels of circRNA [12]. By categorizing samples based on muscle content, we observed that a high muscle content significantly increased circRNA levels. This was further confirmed by our paired analysis of double-extracted samples, where muscle content was the only variable. This finding prompted us to perform linear regression on junction counts as a function of muscle content in order to obtain reliable data throughout the study. Our analysis was carried out on twice as many samples as in the study by Garcia-Rodriguez and coworkers [12] and using an independent approach, including macro-dissection and SEALigHTS. Therefore, our study builds upon and strengthens previous findings. More generally, it shows that muscle content is a confounding factor in circRNA analysis and must be taken into account. Previously published data should be reinterpreted in light of this.

A great diversity in circRNA profiles was found across genes, both in the number of circRNAs identified and their abundance (Table 3). For example, *BARD1* expresses circRNAs that account for up to 80% of its mRNA expression, but most other genes exhibit low circRNA expression, supporting the hypothesis that backsplicing is less efficient than linear splicing [23]. As mRNAs and circRNAs are produced from the same pre-mRNA, our data indicate that genes generating numerous linear splice junctions also tend to generate more circular splice junctions, although this common rule does not apply to all genes (e.g., *ATM* with 71 linear junctions has only 8 circular junctions).

We observed a global decrease in the number of circRNA counts in TT compared to in NT, which is consistent with the existing literature on circRNAs in CRC [8,24], as well as other tumoral pathologies [9,25]. While mRNA reaches equilibrium through rapid synthesis and degradation processes, circRNA has a slow formation rate, and its high stability explains its accumulation in quiescent tissues (nerves and muscles). In actively proliferating cells, circRNA levels may be lower due to a dilution effect, as they are distributed between daughter cells during mitosis. We did not observe any increase in the expression of circRNAs to counterbalance the dilution effect and therefore have no arguments for assigning them a specific role in colorectal tumorigenesis.

Competition between mRNAs and circular RNAs from the host gene is a plausible and attractive mechanism suggested by a previous publication [8]. To demonstrate such a mechanism, we further investigated the dynamics of the circ/lin ratio at the gene level. The variation in this ratio between NT and TT was never associated with an inverse relationship between circular and linear junction counts (i.e., an increase in circular junctions with a decrease in linear junctions, or vice versa). We therefore have no direct evidence of competition between circular and linear RNA production from the same pre-mRNA.

In the MSI group, the absence of significant differences in circ/lin ratios or circular junction counts between normal and tumor tissues shows that mRNAs and circRNA are linked by a similar mechanism but their dynamics differ. Conversely, the MSS group exhibited multiple variations in circ/lin ratios and circular junction counts. The identification of five MSS groups illustrates the variety of regulatory mechanisms or circRNA dysregulation at work in MSS that is not observed in MSI. Interestingly, our analysis revealed decreases in circular junction counts, even when linear junction counts increased. This is a rare observation, as a positive correlation between circular and linear isoform levels is generally observed, consistent with circRNA synthesis being a circularization event of the linear transcript [5]. This observation may be related to regulatory mechanisms in circRNA that are distinct from those in mRNA and that may be involved in the tumorigenesis process.

No statistically significant differences in circ/lin ratios or circular junction counts were found between the 13 MSI tumor samples and the 21 MSS tumor samples. Thus, the differences observed could be linked to tumor characteristics rather than MSI/MSS status, but a lack of statistical power is an alternative explanation. A previous study on MSS/MSI endometrial cancer [26], consisting of an analysis of 10 MSI and 10 MSS tissues, identified 1083 and 864 differentially expressed circRNAs and mRNAs, respectively, but also emphasized the lack of statistical power due to the small sample size. Consequently, the possibility that MSI and MSS differ at the circRNA level remains, and further studies are required. Although speculative, global dysregulation of circRNA formation could potentially explain the observed differences in profiles between the two groups. A study by van den Berg and coworkers [15] supports this hypothesis by highlighting distinct global circRNA profiles across different consensus molecular subtypes (CMSs) of colorectal cancer. The authors found that a high diversity of circRNAs was associated with favorable disease-free survival and that several circRNAs were specifically linked to MSI and CMS, underlining the potential clinical relevance of circRNAs in colorectal cancer. Our study has some limitations. In terms of the MSS and MSI groups, we specifically selected MSS patients suspected of having a cancer predisposition in order to maximize our potential for discovery. The MSI group comprised heterogeneous entities, which can obscure a true biological signal. Similarly, the samples tested differed in terms of lesion type and tumor stage and location, and these differences were not accounted for in the statistical analyses. We were unable to obtain matched healthy and tumor tissue samples for all patients. Due to the low statistical power of the paired analysis, we treated the samples as independent so that we could include all samples in our analysis. Taken together, these factors may limit the generalizability of the MSI–MSS comparison, which may explain why we did not identify any significant differences between the two groups. Furthermore, we opted for stringent statistical adjustments to avoid type I errors, which may also have contributed to this outcome. To move forward, further studies involving large, unselected cohorts of MSS and MSI tumors are required. A large number of samples would also enable the strategy, which currently targets 48 genes, to be expanded to genome-wide circRNA profiling.

Overall, we report a pioneering original study exploring mRNAs and circRNAs in MSS and MSI CRC. Our results do not suggest that competition between circRNAs and mRNAs could lead to the dysregulation of the expression of the key oncogenic genes that we investigated. Instead, they reveal differences in circRNA/mRNA expression patterns within normal and tumor tissues of both MSS and MSI types, and illustrate the importance of considering circRNAs when characterizing tumor processes, even though it is not possible, at this stage, to distinguish between a potential cause and a consequence.

## Figures and Tables

**Table 1:** Additional circRNAs used for the muscle content analysis, including descriptions of parental genes and involved exons. CircBase IDs are provided when the circRNA is listed in the database [13].

circRNA	Parental Gene	Circbase ID	Circularized Exon
circCAMSAP1	*CAMSAP1*	hsa_circ_0001900	exon 2–exon 3
circXPO1	*XPO1*	hsa_circ_0001016	exon 3–exon 4
CircMET	*MET*	hsa_circ_0082002	exon 2
circFMN2	*FMN2*	hsa_circ_0005100	exon 8–exon 13
CiRS-7	*CDR1*	hsa_circ_0001946	after 5′UTR–before 3′UTR
circYAP	*YAP1*	/	exon 2–exon 7
circPPP1R12A	*PPP1R12A*	hsa_circ_0000423	intron 20–exon 22
circERBIN	*ERBB2IP*	hsa_circ_0001492	exon 2–exon 4
circFNDC3B	*FNDC3B*	hsa_circ_0006156	exon 5–exon 6
circZNF609	*ZNF609*	hsa_circ_0000615	exon 2
circSMARCA5	*SMARCA5*	hsa_circ_0001445	exon 15–exon 16
circHIPK3	*HIPK3*	hsa_circ_0000284	exon 2
circPVT1	*PVT1*	hsa_circ_0001821	non-coding RNA 9 exons
circCCDC66	*CCDC66*	hsa_circ_0001313	exon 8–exon 10

**Table 2:** Summary of sample characteristics used for NT-TT and MSI-MSS comparisons. Samples containing more than 50% muscle tissue were removed from the study and are therefore not shown (see the Section 3 for details). The T stage for adenocarcinoma is defined according to the TNM classification [14]. The RNA concentration for each extract was determined using a fluorometric assay and the Qubit^®^ RNA HS (Thermo Fisher Scientific, Waltham, MA, USA), following the manufacturer’s recommendations.

Group	Sample ID	Patient	Nature	Localization	Tissue	Stade T	% Tumoral Cells	% Muscle Cells	Concentration (ng/µL)
MSS	189044TT	4	Colectomy	Left colon	Adenocarcinoma	4	>50	15–25	26.4
MSS	1922210TT	7	Polyp resection	Sigmoid	Adenocarcinoma	/	25–50	<5	28.4
MSS	2011124TT	9	Colectomy	Left colon	Adenocarcinoma	3	>50	25–50	127
MSS	20953TT	12	Colectomy	Left colon	Adenocarcinoma	2	25–50	5–15	41.3
MSS	2111104BISTT	14	Proctectomy	Rectum	Adenocarcinoma	3	25–50	15–25	18.7
MSS	2114811TT	15	Proctectomy	Rectum	Adenocarcinoma	2	25–50	25–50	329
MSS	2116996TT	16	Colectomy	Left colon	Adenocarcinoma	3	25–50	25–50	31.2
MSS	2119227TT	17	Proctectomy	Rectum	Adenocarcinoma	4	25–50	25–50	47.3
MSS	212509TT	18	Polyp resection	Right colon	Adenoma		25–50	<5	16.4
MSS	2211338BISTT	21	Colectomy	Left colon	Adenocarcinoma	4	>50	5–15	36.8
MSS	2213063TT	22	Colectomy	Right colon	Adenocarcinoma	3	>50	15–25	142
MSS	2218412TT	23	Proctectomy	Rectum	Adenocarcinoma	3	25–50	15–25	373
MSS	22519BISTT	24	Colectomy	Right colon	Adenocarcinoma	3	25–50	15–25	74.6
MSS	233745TT	29	Proctectomy	Rectum	Adenocarcinoma	4	>50	15–25	167
MSS	23G1508TT	31	Biopsy	Sigmoid	Adenoma		>50	<5	53.3
MSS	23G1568TT	32	Polyp resection	Rectum	Adenoma		>50	<5	44
MSS	23G1657TT	33	Biopsy	Left colon	Adenocarcinoma	/	25–50	<5	20.6
MSS	23G1926BBISTT	34	Biopsy	Unknown	Adenoma		>50	<5	77
MSS	23G1926CBISTT	34	Biopsy	Unknown	Adenocarcinoma	/	25–50	5–15	32,2
MSS	23G2257TT	35	Colectomy	Left colon	Adenocarcinoma	3	>50	15–25	288
MSS	23G2261TT	36	Colectomy	Left colon	Adenocarcinoma	3	>50	5–15	232
MSS	2011124NT	9	Colectomy	Left colon	NT			25–50	35.3
MSS	20953BISNT	12	Colectomy	Left colon	NT			<5	9.86
MSS	2111104BISNT	14	Proctectomy	Rectum	NT			5–15	8.8
MSS	2114811NT	15	Proctectomy	Rectum	NT			5–15	84.6
MSS	2116996BISNT	16	Colectomy	Left colon	NT			<5	24.4
MSS	2119227BISNT	17	Proctectomy	Rectum	NT			<5	55.2
MSS	212509NT	18	Polyp resection	Right colon	NT			<5	5.39
MSS	2211338BISNT	21	Colectomy	Left colon	NT			<5	25.4
MSS	2213063BISNT	22	Colectomy	Right colon	NT			<5	15.8
MSS	2218412NT	23	Proctectomy	Rectum	NT			15–25	38.4
MSS	22519BISNT	24	Colectomy	Right colon	NT			<5	15
MSS	236034BISNT	30	Proctectomy	Rectum	NT			<5	27.4
MSS	23G1657NT	33	Biopsy	Left colon	NT			<5	12.7
MSS	23G1926ABISNT	34	Biopsy	Unknown	NT			<5	6.38
MSI	169968MSITT	1	Colectomy	Left colon	Adenocarcinoma	4A	>50	<5	70.4
MSI	176833MSITT	2	Polyp resection	Unknown	Adenoma		>50	15–25	116
MSI	1820760MSITT	3	Colectomy	Right colon	Adenocarcinoma	3	>50	25–50	79
MSI	1912697MSITT	5	Biopsy	Right colon	Adenocarcinoma	/	>50	<5	18.9
MSI	1918531MSITT	6	Colectomy	Left colon	Adenocarcinoma	1	25–50	<5	8.04
MSI	2011085MSITT	8	Biopsy	Right colon	Adenocarcinoma	/	>50	5–15	24.8
MSI	205311MSITT	11	Proctectomy	Rectum	Adenocarcinoma	3	>50	<5	98.2
MSI	212613MSITT	19	Colectomy	Right colon	Adenocarcinoma	2	25–50	<5	11.8
MSI	21537MSITT	20	Colectomy	Right colon	Adenocarcinoma	3	>50	5–15	52
MSI	227038MSITT	26	Biopsy	Right colon	Adenocarcinoma	/	15–25	<5	55
MSI	227551MSITT	27	Colectomy	Right colon	Adenocarcinoma	4A	>50	15–25	67
MSI	229240MSITT	28	Colectomy	Right colon	Adenocarcinoma	3	>50	15–25	37.4
MSI	23P21719MSITT	37	Polyp resection	Unknown	Adenoma		>50	5–15	26.4
MSI	169968MSINT	1	Colectomy	Left colon	NT			<5	15.5
MSI	176833MSINT	2	Polyp resection	Unknown	NT			<5	77
MSI	1820760MSINT	3	Colectomy	Right colon	NT			<5	22.6
MSI	1912697MSINT	5	Biopsy	Right colon	NT			<5	5.68
MSI	1918531MSINT	6	Colectomy	Left colon	NT			<5	24
MSI	2011085MSINT	8	Biopsy	Right colon	NT			5–15	33.6
MSI	2013704MSINT	10	Colectomy	Right colon	NT			<5	44.8
MSI	205311MSINT	11	Proctectomy	Rectum	NT			<5	40.6
MSI	2110341MSINT	13	Colectomy	Right colon	NT			5–15	23.8
MSI	212613MSINT	19	Colectomy	Right colon	NT			<5	19.8
MSI	21537MSINT	20	Colectomy	Right colon	NT			5–15	14.7
MSI	225882MSINT	25	Proctectomy	Rectum	NT			5–15	53
MSI	227038MSINT	26	Biopsy	Right colon	NT			<5	49.2
MSI	227551MSINT	27	Colectomy	Right colon	NT			<5	40.8
MSI	229240MSINT	28	Colectomy	Right colon	NT			5–15	35.2
MSI	23P21719MSINT	37	Polyp resection	Unknown	NT			5–15	23

**Table 3:** Characteristics of circular and linear junctions for each gene, ranked by the relative expression of circRNAs to mRNA. Linear junctions include both canonical and alternative junctions. The circRNA/mRNA ratio is calculated for each gene by dividing the sum of UMIs for all circular junctions by mRNA abundance. For example, *BARD1* has the highest circRNA/mRNA expression level at 71.73%, i.e., a relative abundance of 72 circRNAs per 100 mRNA molecules.

Gene	Reference Transcript	Exon Number	Linear Junction Number	Circular Junction Number	circRNAs/mRNA
***BARD1***	NM_000465.4	11	15	14	71.73%
***RAD51D***	NM_002878.4	10	49	24	62.79%
***CHEK2***	NM_007194.4	15	38	22	32.60%
***CCSER2***	NM_001284240.2	10	19	9	32.08%
***ATM***	NM_000051.4	63	71	8	31.88%
***PALB2***	NM_024675.4	13	40	29	23.51%
***BRCA1***	NM_007294.4	23	46	14	22.02%
***BRIP1***	NM_032043.3	20	41	9	17.16%
***MSH3***	NM_002439.5	24	46	24	17.13%
***POLD1***	NM_002691.4	27	37	3	15.13%
***RIC8B***	NM_001330145.2	10	28	9	14.15%
***RAD51C***	NM_058216.3	9	48	12	12.66%
***POLE***	NM_006231.4	49	69	18	11.44%
***RAD51B***	NM_133510.4	11	32	4	7.48%
***MUTYH***	NM_001048174.2	16	37	11	6.24%
***FAM175A***	NM_139076.3	9	20	5	6.09%
***FANCM***	NM_020937.4	23	35	4	5.92%
***MRE11***	NM_005591.4	20	29	5	5.10%
***BMPR1A***	NM_004329.3	13	25	10	5.03%
***BUB1***	NM_004336.5	25	32	6	4.75%
***SMAD4***	NM_005359.6	12	17	9	3.33%
***BRCA2***	NM_000059.4	27	35	3	3.27%
***FAN1***	NM_014967.5	15	23	3	2.39%
***RINT1***	NM_021930.6	15	28	5	2.27%
***APC***	NM_000038.5	16	35	7	2.13%
***CDH1***	NM_004360.5	16	49	31	2.00%
***EPCAM***	NM_002354.3	9	19	10	1.82%
***NBN***	NM_002485.5	16	34	4	1.81%
***MLH1***	NM_000249.4	19	39	4	1.59%
***MSH6***	NM_000179.3	10	16	2	1.59%
***RAD50***	NM_005732.4	25	40	7	1.21%
***MSH2***	NM_000251.3	16	24	2	1.14%
***RNF43***	NM_017763.6	10	15	1	0.84%
***AXIN2***	NM_004655.4	11	18	5	0.76%
***BAP1***	NM_004656.4	17	20	2	0.53%
***POT1***	NM_015450.3	19	28	1	0.46%
***KRAS***	NM_004985.5	5	10	2	0.27%
***STK11***	NM_000455.5	10	17	2	0.26%
***MBD4***	NM_001276270.2	8	12	1	0.09%
***TP53***	NM_000546.6	11	15	1	0.08%
***GALNT12***	NM_024642.5	10	16	1	0.03%
***GREM1***	NM_013372.7	2	3	0	0.00%
***NRAS***	NM_002524.5	7	8	0	0.00%
***NTHL1***	NM_002528.7	6	11	0	0.00%
***PTEN***	NM_000314.8	9	11	0	0.00%
***RPS20***	NM_001023.4	4	5	0	0.00%
***TBP***	NM_003194.5	8	10	0	0.00%
***XRCC2***	NM_005431.2	3	3	0	0.00%

**Table 4:** List of genes with significant differences in expression between normal and tumor tissues in the MSI group, estimated from UMI counts of circular and linear junctions. An arrow, whether up or down, indicates the direction of the change, with FDR-corrected *p*-values from linear regression adjusted for muscle content. NT: normal tissue; TT: tumor tissue; NS: not significant.

Gene	Ratio UMI Count circ/lin Differential Expression NT → TT FDR *p*-Value	Circular Junction UMI Count Differential Expression NT → TT FDR *p*-Value	Linear Junction UMI Count Differential Expression NT → TT FDR *p*-Value
***AXIN2***	NS	NS	↗ *p* = 0.0007
***RNF43***	NS	NS	↗ *p* = 0.00109
***BRCA1***	NS	NS	↗ *p* = 0.00356
***BUB1***	NS	NS	↗ *p* = 0.00356
***CHEK2***	NS	NS	↗ *p* = 0.00356
***BRCA2***	NS	NS	↗ *p* = 0.0387
***TP53***	NS	NS	↗ *p* = 0.04552
***GALNT12***	NS	NS	↘ *p* = 0.01721
***CDH1***	NS	NS	↘ *p* = 0.02415
***APC***	NS	NS	↘ *p* = 0.04234

**Table 5:** List of genes with significant differences in expression between normal and tumor tissues in the MSS group, estimated from UMI counts of circular and linear junctions. An arrow, whether up or down, indicates the direction of the change, with FDR-corrected *p*-values from linear regression adjusted for muscle content. NT: normal tissue; TT: tumor tissue; NS: not significant. NA: not analyzed (no circular RNA).

Gene	Ratio UMI Count circ/lin Differential Expression NT → TT FDR *p*-Value	Circular Junction UMI Count Differential Expression NT → TT FDR *p*-Value	Linear Junction UMI Count Differential Expression NT →TT FDR *p*-Value
***FAM175A***	↘ *p* = 0.00001	↘ *p* = 0.00014	NS
***POLD1***	↘ *p* = 0.00023	↘ *p* = 0.00597	NS
***FAN1***	↘ *p* = 0.00129	↘ *p* = 0.00262	NS
***MSH2***	↘ *p* = 0.00409	↘ *p* = 0.0242	NS
***MSH3***	↘ *p* = 0.00902	↘ *p* = 0.00783	NS
***AXIN2***	↘ *p* = 0.00295	NS	↗ *p* = 0.00025
***RNF43***	↘ *p* = 0.00791	NS	↗ *p* = 0.00004
***BMPR1A***	↘ *p* = 0.00015	↘ *p* = 0.0002	↘ *p* = 0.02368
***ATM***	↘ *p* = 0.00222	↘ *p* = 0.00013	↘ *p* = 0.03164
***MRE11***	↘ *p* = 0.00026	NS	NS
***BRIP1***	↘ *p* = 0.00129	NS	NS
***BARD1***	↘ *p* = 0.00182	NS	NS
***CHEK2***	↘ *p* = 0.00563	NS	NS
***APC***	NS	↘ *p* = 0.01035	↘ *p* = 0.00025
***SMAD4***	NS	↘ *p* = 0.00048	↘ *p* = 0.00163
***MBD4***	NS	↘ *p* = 0.02327	NS
***RIC8B***	NS	↘ *p* = 0.01187	NS
***KRAS***	NS	↘ *p* = 0.01911	NS
***MLH1***	NS	↘ *p* = 0.02391	NS
***CCSER2***	NS	↘ *p* = 0.01035	NS
***XRCC2***	NA	NA	↗ *p* = 0.00929
***NTHL1***	NA	NA	↗ *p* = 0.03878

## Data Availability

The data presented in this study are available in Supplemental Table S5.

## Supplementary Materials

The following supporting information can be downloaded at: https://www.mdpi.com/article/10.3390/genes17091091/s1.
