# Supplementary material for: Unraveling Circular and Messenger RNA Dynamics in Colorectal Tumorigenesis: Insights into Tissue Heterogeneity and MSI-MSS Tumor Distinction
Source: Genes (Basel). 2026 Sep 10;17(9):1091. doi: 10.3390/genes17091091 (PMC13606699; doi:10.3390/genes17091091)
Supplement: Supplementary file 1 [file genes-17-01091-s001.zip › Vautier et al. Supplemental Table 1_revised.pdf]

Supplemental Table 1. Description of the selection criteria for patients suspected of hereditary CRC

**Inclusion: one of the following criteria**
• **CRC in two first-degree relatives, one being diagnosed before 61 years of age**<br>• **CRC diagnosed before 51 years of age or advanced colorectal adenoma (diameter over one cm, and/or tubulovillous or villous and/or with high-grade dysplasia) before 41 years of age**<br>• **Multiple primary CRCs in the same individual, the first one being diagnosed before 61 years of age**
**Exclusion criteria**
• **Lynch syndrome, as defined by the presence in the patient of a germline MMR gene pathogenic variant and/or a MSI tumor, in the context of a suggestive presentation (familial history, early age of onset)**<br>• **Adenomatous polyposis, as defined by more than 10 histologically proven adenomas**<br>• **Hamartomatous polyposis, as defined by the presence of histologically proven hamartomas**
