# Supplementary material for: Unraveling Circular and Messenger RNA Dynamics in Colorectal Tumorigenesis: Insights into Tissue Heterogeneity and MSI-MSS Tumor Distinction
Source: Genes (Basel). 2026 Sep 10;17(9):1091. doi: 10.3390/genes17091091 (PMC13606699; doi:10.3390/genes17091091)
Supplement: Supplementary file 1 [file genes-17-01091-s001.zip › Vautier et al. Supplemental Table 2_revised.pdf]

Supplemental Table 2. Muscle content for samples from both extractions

Sample	Muscle content Extract 1	Muscle content Extract 2
20953NT	25-50%	<5%
2111104NT	25-50%	5-15%
2111104TT	25-50%	15-25%
2116996NT	>50%	<5%
2119227NT	25-50%	<5%
2211338NT	>50%	<5%
2211338TT	25-50%	5-15%
2213063NT	25-50%	<5%
2218601NT	25-50%	<5%
236034NT	25-50%	<5%
