# Supplementary material for: Unraveling Circular and Messenger RNA Dynamics in Colorectal Tumorigenesis: Insights into Tissue Heterogeneity and MSI-MSS Tumor Distinction
Source: Genes (Basel). 2026 Sep 10;17(9):1091. doi: 10.3390/genes17091091 (PMC13606699; doi:10.3390/genes17091091)
Supplement: Supplementary file 1 [file genes-17-01091-s001.zip › Vautier et al. Supplemental Table 3_revised.pdf]

Supplemental Table 3. Quality metrics.

The main quality-control metric we use for SEALigHTS analysis is the proportion of probes that hybridize correctly at exon-exon junctions. Our in-house Python script quantifies the percentage of reads corresponding to probes that are correctly ligated and identified on both sides of the junction (% Left and Right OK -> % LR OK). All samples included in this study had a % LR OK above 20%. Based on empirical observation, we adopted this value as a practical quality indicator: manual inspection of samples with a % LR OK below 20% showed that detection of the canonical junctions, *i.e.* those corresponding to linear splicing of the MANE Select transcript, was no longer reliable. Therefore, a % LR OK above 20% provides reasonable assurance that the RNA is of sufficient quality for SEALigHTS analysis. The total number of unique molecular identifiers (UMI) is indicated for each sample. The amplification factor was obtained by dividing the total number of reads by the total number of UMI for each sample.

Sample ID	% L+R OK	UMI	amplification factor
169968MSITN	0.38	238818	6.81
169968MSITT	0.43	262720	10.32
176833MSITN	0.48	272677	8.6
176833MSITT	0.47	301263	14.27
1820760MSITN	0.41	206381	12.93
1820760MSITT	0.56	477153	7.55
189044TT	0.57	478626	11.26
1912697MSITN	0.36	200525	8.83
1912697MSITT	0.68	577047	8.74
1918531MSITN	0.61	506567	11.91
1918531MSITT	0.35	151288	17.28
1922210TT	0.71	852708	8.2
2011085MSITN	0.79	693461	9.11
2011085MSITT	0.78	803339	8.52
2011124TN	0.67	793894	7.43
2011124TT	0.74	1014887	6.96
2013704MSITN	0.74	661905	7.58
205311MSITN	0.7	713357	12.71
205311MSITT	0.71	718601	9.15
20953BISTN	0.4	226354	9.57
20953TT	0.65	628763	10.23
2110341MSITN	0.65	381876	16.05
2111104BISTN	0.44	247072	15.00
2111104BISTT	0.65	540494	10.87
2114811TN	0.79	1183347	6.25
2114811TT	0.72	759575	7.99
2116996BISTN	0.55	499578	11.48
2116996TT	0.65	922298	5.89
2119227BISTN	0.78	854631	12.6
2119227TT	0.76	1138534	5.63
212509TN	0.45	340184	14.44

212509TT	0.76	1009806	6.14
212613MSITN	0.67	625553	12.81
212613MSITT	0.41	265685	10.04
21537MSITN	0.27	112010	15.43
21537MSITT	0.43	292281	11.5
2211338BISTN	0.68	592560	6.74
2211338BISTT	0.6	506606	7.5
2213063BISTN	0.57	313258	12.95
2213063TT	0.81	1214001	7.02
2218412TN	0.69	905831	6.8
2218412TT	0.81	1591710	3.82
22519BISTN	0.46	240505	17.57
22519BISTT	0.53	288641	14.55
225882MSITN	0.82	860322	10.3
227038MSITN	0.84	1222097	6.11
227038MSITT	0.84	1128137	7.24
227551MSITN	0.8	767456	8.75
227551MSITT	0.69	680348	12.02
229240MSITN	0.8	687999	11.92
229240MSITT	0.24	109308	14.47
233745TT	0.86	1235940	5.07
236034BISTN	0.41	297190	12.67
23G1508TT	0.89	2061497	4.48
23G1568TT	0.82	1508897	5.61
23G1657TN	0.81	1528565	5.7
23G1657TT	0.75	1728457	3.68
23G1926ABISTN	0.58	426709	11.88
23G1926BBISTT	0.32	145537	24.83
23G1926CBISTT	0.85	1512248	6.06
23G2257TT	0.83	1307254	6.22
23G2261TT	0.89	1110579	8.87
23P21719MSITN	0.85	1448221	5.45
23P21719MSITT	0.9	922432	9.29
