# Supplementary material for: Unraveling Circular and Messenger RNA Dynamics in Colorectal Tumorigenesis: Insights into Tissue Heterogeneity and MSI-MSS Tumor Distinction
Source: Genes (Basel). 2026 Sep 10;17(9):1091. doi: 10.3390/genes17091091 (PMC13606699; doi:10.3390/genes17091091)
Supplement: Supplementary file 1 [file genes-17-01091-s001.zip › Vautier et al.Supplemental Table 4_revised.pdf]

Supplemental Table 3. List of genes with significant differences in expression between normal and tumor tissues, estimated from UMI counts of circular and linear junctions. An arrow, whether up or down, indicates the direction of the change, with FDR-corrected p-values from linear regression adjusted for muscle content. NT: normal tissue. TT: tumor tissue.

Gene	Linear junctions UMIs counts<br>Differential expression<br>NT →TT<br>FDR p-value
RNF43	↗ p = *0,000001*
AXIN2	↗ p = *0,000004*
XRCC2	↗ p = *0,00051*
BRCA1	↗ p = *0,00078*
CHEK2	↗ p = *0,00078*
NTHL1	↗ p = *0,00078*
BUB1	↗ p = *0,00111*
BRIP1	↗ p = *0,00207*
BRCA2	↗ p = *0,00559*
MSH2	↗ p = *0,02825*
PALB2	↗ p = *0,03452*
TP53	↗ p = *0,04728*
APC	↘ p = *0,000003*
SMAD4	↘ p = *0,00034*
BMPR1A	↘ p = *0,00144*
GALNT12	↘ p = *0,00161*
PTEN	↘ p = *0,0169*
ATM	↘ p = *0,01863*
CDH1	↘ p = *0,04024*
EPCAM	↘ p = *0,04024*
